# Danger Signals in Cardiovascular Disease

**DOI:** 10.1155/2014/395278

**Published:** 2014-04-06

**Authors:** Stefan Frantz, Claudia Monaco, Fatih Arslan

**Affiliations:** ^1^Department of Internal Medicine I, University Hospital Würzburg, Comprehensive Heart Failure Center, University of Würzburg, Oberdürrbacher Straße 6, 97080 Würzburg, Germany; ^2^Comprehensive Heart Failure Center, University of Würzburg, Germany; ^3^Kennedy Institute of Rheumatology, University of Oxford, 65 Aspenlea Road, London W6 8LH, UK; ^4^Laboratory of Experimental Cardiology, University Medical Center Utrecht, The Netherlands; ^5^Department of Cardiology, University Medical Center Utrecht, Heidelberglaan 100 Room G.02.523, 3584 CX Utrecht, The Netherlands

More than ten years ago, the “danger theory” challenged conservative immunology. At that time, the consensus was that the immune system is activated by antigens recognized as nonself. However, the self-nonself theory gave no explanation why, for example, a fetus with obvious foreign antigens does not lead to maternal immune activation whereas transplanted organs do. In an attempt to resolve these apparent paradoxes, the danger theory postulated that the immune system is triggered by “danger signals” released upon tissue injury and stress alerting the immune system that there is risk to the host [1]. The “danger theory” is supported by the growing number of endogenous ligands that can activate innate immune receptors such as toll-like receptors, RIG-I-like receptors, NOD-like receptors, and the inflammasome. However, it poses the challenge of identifying such signals and the mechanisms of their generation rigorously. Danger signals or danger associated molecular patterns (DAMPs) identified so far include factors like high-mobility group protein B1, mitochondrial DNA, heat shock protein (HSP), interleukin-1*α*, adenosine triphosphate, reactive oxygen intermediates, and uric acid [2].

A common feature of cardiovascular diseases, like myocardial infarction, heart failure, atherosclerosis, and so forth, is a robust inflammatory response. The reason for an immunologic reaction in mostly nonimmune diseases is not very well defined. However, the danger theory offers a good explanation: tissue damage, for example, in myocardial infarction, could lead to the release of danger signals and thereby cause an immune response. Indeed, in the current issue, several aspects of this process are highlighted: after a general introduction into DAMPs in the cardiovascular system [3], M. Ashri et al. discuss the theory of cardiotrophin-1 as a secondary DAMP in obesity “*Update on the pathophysiological activities of the cardiac molecule cardiotrophin-1 in obesity*,” whereas A. Schiopu et al. review S100A8 and S100A9, members of the calgranulin family, as potential DAMP in cardiovascular disease “*S100A8 and S100A9*:* DAMPs at the crossroads between innate immunity, traditional risk factors, and cardiovascular disease*.” F. van den Akker et al. highlight that danger signals might influence the phenotype of mesenchymal stem cells and secondarily outcome after myocardial infarction “*Mesenchymal stem cell therapy for cardiac inflammation: immunomodulatory properties and the influence of toll-like receptors*.” A few original articles deal with the role of oxidative stress as DAMP “*Berberine protects against palmitate-induced endothelial dysfunction: involvements of upregulation of AMPK and eNOS and downregulation of NOX4*” and “*Natural antioxidant-isoliquiritigenin ameliorates contractile dysfunction of hypoxic cardiomyocytes via AMPK signaling pathway*” and with actin or chitinase 3-like 1 as a trigger of immune activation in patients with advanced atherosclerotic plaques “*Actin is a target of T-cell reactivity in patients with advanced carotid atherosclerotic plaques”* and “*Increased expression of chitinase 3-like 1 in aorta of patients with atherosclerosis and suppression of atherosclerosis in apolipoprotein E-knockout mice by chitinase 3-like 1 gene silencing*” or complement factor C3 as marker of danger signal activation in patients with heart failure “*Complement c3c as a biomarker in heart failure*.” All manuscripts underline the importance of danger signals in cardiovascular disease in basic as well as clinical science.

*Clinical Implications*. DAMPs may have great diagnostic, prognostic, and therapeutic potential. In theory, DAMPs may indicate active tissue injury. Since DAMP levels are related to the extent of injury, they may have prognostic implications. When DAMPs are the most important trigger for immune activation, pharmaceutical interference should allow tailoring an immune response. However, it has to be beard in mind that the activation of the immune system in the context of tissue injury makes evolutionary sense and is not necessarily negative. For example, after myocardial infarction depletion of macrophages causes the scar not to be cleared of cell debris and left ventricular thrombi to develop leading to adverse outcome in animals and potentially also in humans (*Monocytes/macrophages prevent healing defects and left ventricular thrombus formation after myocardial infarction*). Thus, an initial immune activation is necessary for a coordinated pathophysiologic and beneficial response to injury. However, a chronic immune activation might be detrimental, as has been shown by several groups. Therefore, timing will be crucial when interfering with DAMPs.

In conclusion, a better understanding of DAMPs in cardiovascular disease might give us dual benefit: it will help us to identify and treat patients at the very core of the pathophysiological process. However, markers and potential drug targets warrant further research.

*Stefan Frantz* *Stefan Frantz* *Claudia Monaco* *Claudia Monaco* *Fatih Arslan* *Fatih Arslan*
